# Flourishing at the end of life

**DOI:** 10.1007/s11017-024-09679-x

**Published:** 2024-08-20

**Authors:** Xavier Symons, John Rhee, Anthony Tanous, Tracy Balboni, Tyler J. VanderWeele

**Affiliations:** 1 Human Flourishing Program in the Institute for Quantitative Social Science, Harvard University, Cambridge, MA USA; 2 Plunkett Centre for Ethics, The Australian Catholic University, Sydney, NSW Australia; 3 Department of Epidemiology, TH Chan School of Public Health, Harvard University, Cambridge, MA USA; 4 Center for Neuro-oncology, Department of Medical Oncology, Dana Farber Cancer Institute, Boston, MA USA; 5 School of Medicine, University of New South Wales, Sydney, NSW Australia; 6 Division of Adult Palliative Care, Department of Psychosocial Oncology & Palliative Care, Dana Faber Cancer Institute, Boston, MA USA; 7 Department of Neurology, Harvard Medical School, Boston, MA USA

**Keywords:** Wellbeing, Eudaimonia, Aristotle, Terminal illness, Death, End of life, Flourishing

## Abstract

Flourishing is an increasingly common construct employed in the study of human wellbeing. But its appropriateness as a framework of wellbeing at certain stages of life is contested. In this paper, we consider to what extent it is possible for someone to flourish at the end of life. People with terminal illness often experience significant and protracted pain and suffering especially when they opt for treatments that prolong life. Certain aspects of human goods, however, that are plausibly constitutive of flourishing—such as meaning and purpose, deep personal relationships, and character and virtue—can be uniquely realised when life is ending. We argue that there is a qualified sense in which one can flourish at the end of life but that one must make important modifications to the criteria implicit in conventional conceptions of flourishing. We close with a discussion of the empirical assessment of wellbeing at the end of life and explore the possibility of introducing a flourishing measure in palliative care practice.

## Introduction

Flourishing is an increasingly popular construct in wellbeing studies. But its appropriateness as a construct for measuring wellbeing at different stages of human life is contested [1, 2]. To what extent can one *flourish* when living with a terminal illness? On the one hand, people with terminal illness would appear to have significant constraints on their quality of life. Terminal illness involves a downward trend in health and ends with death. It may include significant pain and suffering (we understand pain as referring to a sensory experience associated with, or resembling that associated with, actual or potential tissue damage; we understand suffering to be an undesired experience, of considerable duration or intensity, of a negative physical or affective state and that may involve a loss of a person’s sense of self) [3–6]. These hallmarks of the final chapters of life may lead one to conclude that flourishing is not possible when life is ending and that the language of flourishing is naïve and inappropriate in end of life contexts.

On the other hand, some criteria commonly taken to be constitutive of human flourishing—such as meaning and purpose, deep personal relationships, and character and virtue [7–9] – may be attainable even in one’s final days. One might even argue that these aspects of a good life are, in fact, in some ways *uniquely realizable* at the end of life. Ill-health and dying can paradoxically enable a special realization of certain aspects of a flourishing life. This is not to say that everyone—or even the majority of people—experience a strong sense of meaning and purpose, deeper social connectedness, or moral growth at the end of life. But some may [10, 11]. This fact seems worthy of closer examination than it has received to date.

The topic is particularly important given that a good death is arguably an important part of a flourishing life [12]. Conversely, a tragic or agonizing death can mar an otherwise good life [13, p. 1100a6–8]. One could argue, therefore, that wellbeing at the end of life has a special importance in the context of the assessment and promotion of human flourishing.

In this article, we consider how the end of life presents special opportunities for moral growth and may be uniquely conducive to the cultivation of meaning and purpose and deep personal relationships. Specifically, we explore the contention that the existential context of the final months of life may allow one to consolidate one’s life narrative, cultivate virtue, and deepen and bring to completion one’s close social relationships in a way that may not be as feasible in other contexts in life. Drawing upon these insights, we explore the possibility of introducing a flourishing measure in palliative care. Terminally ill patients, by definition, have low physical health-related wellbeing. With these and other considerations in mind, we suggest that assessments of flourishing at the end of life ought to be interpreted according to the unique circumstances of the final chapter of life. Health, for example, should not be expected to be high in the assessment of flourishing in end-of-life contexts. With these considerations acknowledged, it is possible to develop conditional notions of flourishing to appropriately qualify what aspects of flourishing should be emphasized at the end of life.

In the first section, we introduce flourishing as an approach to the study of wellbeing. In the second, we offer some preliminary remarks about the evaluation of human flourishing. In the third, we discuss the possibility of flourishing at the end of life, while in the fourth we discuss current approaches to the empirical assessment of wellbeing at the end of life. And in the fifth section, we explore the implications of our discussion for the assessment of flourishing in end-of-life contexts, explore the possibility of introducing a flourishing measure in palliative care, and compare and contrast it with current quality-of-life approaches.

## ‘Flourishing’ as an approach to the study of wellbeing

Human flourishing is a concept with a rich philosophical pedigree dating back to the classical era, particularly in Aristotle’s ethical writings. Interest in flourishing has been rekindled with the mid-twentieth century revival of Aristotelian virtue ethics in moral philosophy as well as a renewed interest in character, virtue, and wellbeing in positive psychology [14] and in educational theory [15].

It is instructive to consider characteristics of the construct of flourishing that distinguish it from other contemporary approaches to the study of wellbeing. Flourishing has, at least historically, linked the question of wellbeing to the nature and proper function of a living thing. In the *Nicomachean Ethics*, for example, Aristotle [13] argues that the good of a thing is fundamentally connected with its function and the excellent performance of that function. Thus, in his inquiry into the human good, he observes that:

just as the good, i.e., [doing] well, for a flautist, a sculptor, and every craftsman, and in general, for whatever has a function and [characteristic] action, seems to depend on its function, the same seems to be true for a human being, if a human being has some function [13, p. 1097b25-30].

Aristotle goes on to conclude that the proper function of a human being is rational activity in accord with virtue, and that flourishing, for humans, consists in excellent use of human reason in both practical and speculative matters.

A similar view has been advanced by neo-Aristotelian scholars. Philippa Foot, for example, develops the view in *Natural Goodness* [7] that what is good for a human being is at least partially informed by the features of a human form of life. Foot defines natural goodness as “intrinsic or ‘autonomous’ goodness in that it depends directly on the relation of an individual to the ‘life form’ of its species” [7, pp. 26–27]. While human rationality has the capacity to assess different reasons for action—and is thus not pre-determined by nature—it is also not the case that well-ordered reason operates in a vacuum. Rather, it takes as its point of departure certain facts about human life and the human condition and reasons based on these facts about how one ought to act. One cannot understand human rationality and wellbeing without a prior reference to the nature and function of human life, or so it is argued [7, ch. 4]. In the positive psychology literature, similarly, the term “flourishing” has been used in conceptions of well-being that move beyond happiness and life satisfaction [14, 16–18] and sometimes make explicit reference to the fulfilment of human potential in conceptualization and measurement proposals [18].

Flourishing tracks the development of a living thing and has as its paradigm the healthy and vigorous biological development of an organism over time. It is indexed to the ‘well-functioning’ of a living thing and that well-functioning is something that is evidenced across the course of time rather than just during particular moments of its existence [19, 20]. Flourishing, then, is primarily concerned with the whole life of an organism rather than just a particular moment in the life of that organism. Aristotle himself notes that “one swallow does not make a spring, nor does one day; nor, similarly, does one day or a short time make us blessed or happy” [13, p. 1098a18-20]. Instead, one must live out rational activity in accord with virtue across a ‘complete life’ if one is to be happy.

This is not to say that assessments of flourishing are impossible at discrete stages in a person’s life. The word flourishing is often used in everyday language with reference to particular stages of a person’s life. Someone might remark, for example, that they “only really flourished at college,” or “I only flourished when I moved to New York.” It is also true that one might use the language of flourishing to refer to a particular aspect or other of a person’s life—e.g., a flourishing career or a flourishing marriage. One need not, therefore, limit one’s understanding of flourishing to the whole life of a person. Furthermore, the adjective ‘flourishing’ can be used in a time-specific and domain-specific manner.

But given the teleological assumptions built into the Aristotelian concept of flourishing, flourishing in its broadest context ought to be understood with reference to a *flourishing life*. Flourishing is primarily concerned with the attainment of the purposes toward which a life is oriented. Other uses of the word flourishing can be understood as analogous, secondary uses of the term.

One further note on the language of flourishing is in order. Flourishing may sometimes be thought to involve ‘untroubled success’ in life [7]. But such an account of flourishing may be slightly misleading insofar as flourishing is really just a byword for the human good. It would be more accurate to refer to flourishing as a property that is instantiated in differing degrees to the extent that one is participating fully in the goods of human life. Or more simply, one can be said to be flourishing to the extent that they are living an authentically human life, even if one faces a certain amount of adversity. Thus, flourishing may be seen as “the relative attainment of a state in which all aspects of a person’s life are good” [21, p. 37].

## Human flourishing and the end of life: preliminary considerations

Given these considerations, how ought one approach the task of assessing human flourishing? Recently, efforts have been made to explore how a construct of flourishing can be operationalised to measure human wellbeing. VanderWeele [8], for example, has identified six domains of flourishing that are, as a minimum, befitting of inclusion in any attempt to assess the distribution and determinants of human flourishing. These are, namely, happiness and life satisfaction, physical and mental health, meaning and purpose, virtue and character, close social relationships, and financial stability. The first five of these domains are considered ends and the sixth constitutes an important means to sustain many of these ends. VanderWeele’s argument is not that these are exhaustive of what constitutes flourishing but that each is a part of it.

This approach to the study of wellbeing might be thought to capture dimensions of human wellbeing that other constructs in the social sciences, such as hedonic happiness and evaluative happiness on their own, fail to track [8]. Aristotelian flourishing, after all, is distinct from contemporary philosophical theories of wellbeing as well as contemporary accounts of flourishing employed in the social sciences [22]. VanderWeele’s flourishing index focuses on diachronic properties of a life (close social relationships and the cultivation of virtue and character) or properties that pertain to the intelligible structure of a life (meaning and purpose) in addition to subjective evaluations of one’s life (happiness and life satisfaction). These domains reflect the contention that flourishing is concerned with stable, diachronic properties of a life and not just momentary wellbeing. In such a way it bears similarity to the wellbeing constructs in the gerontology literature that include cognitive, affective, behavioral, and experiential domains, with somatic, functional, and other health-related facets and markers of social indicators; but it also extends beyond these to include character and virtue—central in Aristotelian and other conceptualizations of well-being [8, 22].

Flourishing measures such as VanderWeele’s have been employed in a wide variety of contexts. These measures have been used to assess wellbeing across age-groups—from childhood and adolescence [23] to adulthood [24] and in various social, professional, and cultural contexts. Research has also been conducted on *pathways to flourishing*, or “aspects of human life that have relatively large effects” on the different domains of human flourishing [8, p. 8150].

At this juncture, however, we wish to make an initial comment concerning assessments of flourishing at the end of life that helps to illustrate our interest in the use of a flourishing measure in palliative care contexts. This comment concerns the provisional nature of flourishing assessments prior to death and the special importance of a good conclusion to one’s life.

Assessments of lifetime flourishing are inherently provisional until a life has concluded. Flourishing assessments may only be made definitively at the end of life at least insofar as one is focused on the complete-life wellbeing of a person. Assessments prior to this point are always partial and open to revision depending on what comes after the assessment. Indeed, one might experience significant good fortune in one’s final days and have a very propitious end to one’s life despite earlier hardship. Alternatively, one could potentially experience significant *misfortune* at the end of life that might blight an otherwise flourishing life. This latter consideration was one that Aristotle was particularly sensitive to. In the *Nicomachean Ethics*, he noted how great misfortune at the end of life can potentially mar an otherwise flourishing existence. He writes:

[happiness] needs a complete life because life includes many reversals of fortune, good and bad, and the most prosperous person may fall into a terrible disaster in old age, as the Trojan stories tell us about Priam. If someone has suffered these sorts of misfortunes and comes to a miserable end, no one counts him happy [13, p. 1100a6–8].

By way of context, Priam was Troy’s last king and was brutally slain when the city was sacked by the Greeks. One may, in other words, have a life that is for the most part characterized by virtue and suitably furnished with the trappings of success but still experience great misfortune at the end of life and not be considered blessed. Dying a good death, then, appears to be an important and, indeed, constitutive feature of a flourishing life.

It would be wrongheaded to take from this consideration that one should only deploy the concept of a flourishing life when evaluating the lives of deceased persons. As we acknowledged earlier, flourishing is a term that can be used to refer to specific time periods in a person’s life or specific domains of one’s life. Even where assessments of flourishing have these parameters, the construct of flourishing is still more comprehensive than measures of wellbeing that focus on hedonic experience, opportunity, or life satisfaction.

But wellbeing at the end of life must be considered when assessing one’s overall lifetime flourishing, and any assessment of lifetime flourishing that fails to adequately capture wellbeing at the end of life is deficient. Indeed, our point is stronger than this. Our point is, in fact, that one’s flourishing at the end of life has special importance. This is because the shape of the arc of one’s life matters, and one’s lifetime flourishing is not merely an additive function of momentary wellbeing [25, 26]. Nor is it just about ‘adequate’ wellbeing at the end of life [27]. Rather, it seems that lifetime flourishing is partly determined by the narrative structure of one’s life, and a kind of closure or denouement at the conclusion of one’s life [28, 29].^1^ One’s life commitments must come to a kind of fruition in one’s final days if one is truly to be said to have flourished. Note that one can experience great misfortune midlife but then recover and rebuild one’s life such that the overall effect of the misfortune on one’s life as a whole is significantly mitigated, or even seen subsequently as having been altered to a different and ultimately superior trajectory. Misfortune at the end of life, however, is like a tragic end to a story that can definitively change how one understands the very genre of the story.

Interestingly, Aristotle is concerned with material circumstances (e.g., wealth, health, social status) as necessary aspects of flourishing in a way that Christian and post-Christian interpreters of Aristotle often are not [30]. For Aristotle, Priam was materially ruined—his kingdom lost, his son dead—so there was no way he could be flourishing; he had lost the ‘magnanimity’ or ‘magnificence’ that one requires to flourish. However, many later commentators on Aristotle interpret flourishing in a more moral and less material way: i.e., someone can still be flourishing if he or she has moral virtues, even if he or she happens to be poor, sick, part of a lower social class, and so on. It is worth noting, however, that the gerontology literature has shown association between lower education, income, and net worth, and greater impairment and more rapid deterioration in functionality in later life [31]. Material circumstances are not irrelevant. Nevertheless, we aim to show that the end of life still has special importance because it presents unique opportunities for growth in character, close-social relationships, and meaning and purpose.

There is much that could be said on this point, but suffice it to say here that flourishing can only be attributed in a very qualified way to the life of someone who has experienced considerable positive wellbeing throughout the course of their life but then dies a horrible and protracted death wherein the person also has major failures of character and further witnesses the failure of the person’s various relationships or life projects. A good death, in contrast, can positively alter the way one views a life that was otherwise affected by suffering and adversity.

## The possibility of flourishing at the end of life

To what extent, then, is flourishing possible at the end of life? Or, to put the question differently, is flourishing an appropriate construct with which to think about wellbeing at the end of life?

Notwithstanding some of the more sobering observations that one can make about terminal illness and the dying process, this section will consider how some of the goods constitutive of human flourishing may be realized in a uniquely deep way at the end of life. We will focus on three themes in particular: meaning and purpose in the form of narrative integration; close social relationships; and virtue and character. We will explore how these domains of flourishing become especially relevant in end-of-life contexts and how the goods at the center of these domains may in at least some cases be uniquely realized at the end of life.

To reiterate, the aim of this exercise is not to glorify terminal illness or the dying process. Terminal illness may impose certain, perhaps profound, physical and psychological limitations on a person. We do not wish to romanticize the dying process and the pain and suffering that typically accompany it. We also do not deny that many individuals would rate their wellbeing very low at the end of life and that there is perhaps a paradox in the idea that flourishing can occur when a patient’s subjective assessment of pain, suffering, and functionality may be in tension with this. As mentioned in the previous section, flourishing is a multidimensional construct that is intended to track aspects of wellbeing that other measures assessing momentary, material, or purely subjective wellbeing do not track.

But precisely because our focus is on a hybrid, multidimensional account of wellbeing, we would like to put pressure on an assumption that some might hold, namely, that flourishing is impossible or exceedingly rare in the final chapters of life. Ezekiel Emanuel, for example, famously declared in a 2014 article in *The Atlantic* that he hoped to die at 75 [32]. He noted that living too long “renders many of us, if not disabled, then faltering and declining,” “robs us of our creativity and ability to contribute to work, society, the world,” and leaves us “feeble, ineffectual, even pathetic” [32]. One might extrapolate from this the idea that living with a terminal illness is not compatible with the realization of fundamental human goods. In addition to the traits that Emanuel has mentioned, terminal illness can bring with it immense suffering and pain. As such, one might contend that terminal illness, regardless of when it occurs, renders the human person incapable of flourishing.

We wish to challenge this idea, and to instead argue that there are certain dimensions of human existence that one can enter into more fully when one’s life is ending. Rather than being a time of ‘mere oblivion,’ as Shakespeare’s Jaques contends in *As You Like It* [33], we contend that the end of life can be pregnant with meaning and significance for human beings. Indeed, one might suggest that the medieval *Ars Moriendi* (‘Art of Dying’) literature is a more fruitful source of insight into the deep existential challenges, but also moral growth, that one can experience at the end of life [11].

We note further, however, that the subsequent discussion and the proposed questionnaire has limited applicability for people who are cognitively impaired. The three themes of meaning and purpose, close social relationships, and virtue and character will be discussed with reference to people with relatively normal cognitive capacity. We by no means intend to deny that flourishing is possible for people without the cognitive capacity to do the flourishing assessment (e.g., in advanced dementia), but we do think that an adequate treatment of that topic requires a separate study and very nuanced exploration. That exploration should include development of proxy assessments of flourishing (e.g., by a family member), as has been done for many extant palliative care instruments [34, 35] given how common it is for a dying person to be unable to perform assessments [36]. With such a future step in mind, we will herein limit our focus in this study to individuals who have relatively operative cognitive capacity notwithstanding their ill-health or age.^2^

### Meaning and purpose

Flourishing (or, one of its contraries—languishing) at the end of life is contingent on one’s capacity to find meaning when life is ending [37]. One must make sense of the stark truth that one’s life is coming to an end, and, in some cases, in unexpected and distressing circumstances.

Here, it is appropriate to consider the themes of narrative and storytelling. Several philosophers have described human beings as narrative or story-telling creatures [38, 39]. The basic claim is that human beings are creatures with a memory, imagination, emotions, and will and make sense of their lives not simply by comprehending momentary events but rather constructing narratives concerning the nature of those events and the relationship that exists between different events and oneself. This process of meaning-making is an ongoing interpretive process and can be revised over time. This is not to say that the events of one’s life are endlessly open to interpretation and that life events place no truth constraints on the kind of meaning one makes from one’s life. But it is true that one can interpret one’s life in various ways and that, depending on the kind of narrative or lack thereof that one constructs, one’s wellbeing may be significantly affected.^3^

Interpretive self-authorship of this form can be distinguished from what we call agential self-authorship. Agential self-authorship involves action in the world whereby one constructs the base matter of one’s identity and life story. One might, for example, think of the actions whereby one pursues a career in medicine (such as getting a medical degree, participating in a training program, and/or applying for a medical license), or the actions whereby one might start a family (such as finding a partner, getting married, and attempting to conceive). Interpretive self-authorship, on the other hand, is concerned with the way in which one makes sense of one’s life and the events that characterize it. For example, one might judge one’s career in medicine to have been a success based on certain interpretations of facts (e.g., good patient outcomes, professional acclaim, widely cited research, etc.) or one might see one’s family life as happy based on certain interpretations of the facts (e.g., happy and successful children, good memories, etc.). It may be the case that in making such judgements one may choose to focus on certain positive outcomes and to downplay certain negative outcomes. Such interpretive judgements can be distinguished from action in the world whereby one seeks to give additional content to one’s life narrative. Thus, whereas agential self-authorship gives rise to a greater sense of *purpose*, which tends to be more end-directed, interpretative self-authorship gives rise to a greater sense of *meaning*, which tends to focus more on understanding the relations of one’s identity and life events to a greater context [40].

What bearing might this have on the end of life? Some people may find themselves in a situation of existential despair in the face of death—there are many aspects of the dying process that can understandably lead to such a response. To return to Shakespeare’s Jaques, one might describe the end of life as a “second childishness” in which one is reduced to the merest of existences, “sans teeth, sans eyes, sans taste, sans everything” [33, p. 229].

But it is also true that one may acquire a clearer sense of meaning at the end of life. There are ways in which narrative construction may be facilitated by the very fact that one’s life is ending. When someone has a terminal illness, one sometimes knows the parameters of one’s remaining time. One’s life, then, acquires a very determinate time horizon. On the one hand, one might view this to be something that limits one’s capacities for agential self-authorship insofar as one may be limited in one’s ability to refashion one’s life through concrete actions. On the other hand, there is a sense in which one’s capacities for interpretive self-authorship are enhanced insofar as more of one’s life is complete, and moreover one’s life has entered a stage of denouement where there are likely to be fewer and fewer significant events that might otherwise affect one’s life interpretation. One can, in other words, start to make definitive evaluations of one’s life given that the narrative arc of life is nearing its end.

Paradoxically, the process of narrative consolidation may also be facilitated by the fact that people are limited and afflicted in various ways. The fact that someone may have more free time due to no longer being able to work or live an active life, for example, may facilitate a deeper reflection on the narrative contours of one’s life. A particularly poignant example of this might be the life of Morrie Schwatz, a sociology professor from Brandeis University who died in 1995 after a lengthy battle with Amyotrophic Lateral Sclerosis (ALS). Schwartz used his illness as an opportunity to reflect on and find meaning in the experience of dying and to reflect at length on the character and contours of his own life. His life story eventually became the subject of a best-selling memoir by Mitch Albom, *Tuesdays With Morrie* [41].

One should, therefore, acknowledge the unique opportunity for the consolidation of one’s life narrative that often arises at the end of life, and the fact that the end of life may present a unique opportunity to acquire a deeper sense of meaning. People construct meaning in different ways and can respond in diametrically opposed ways to the experience of adversity or suffering. One person’s epiphany may be another’s desolation. But suffering can, for some, be the occasion for acquiring a deeper sense of meaning and purpose. People also appear to become more spiritual as they enter later life [42, 43]. To reiterate, our claim is not that flourishing will necessarily occur in these contexts, but merely that it is *possible*, and, indeed, less exceptional than some may believe.

It should also be noted that the final chapters of life need not entail an absence of possibilities for agential self-authorship. Some people are quite active up until the final days of a terminal illness. In any case, at least some of the constituent elements of a flourishing life—such as close social relationships—still remain accessible to one even when one is otherwise limited by illness. One also retains agency with regard to one’s moral and spiritual identity even at the end of life.

As such, people with terminal illness will often reassess and re-order their priorities as they near the end of their life. When someone knows that they have two, six, or twelve months to live, they often give increased focus to what they deem to be most important. Whereas previously, certain pragmatic goals such as reaching professional performance indicators may have been the focus of one’s energies—even to the detriment of one’s overall wellbeing—when time and perhaps physical and mental capacities are limited, one may focus less on these goals and instead give attention to other goals, such as the cultivation or restoration of close familial relationships or spiritual and religious engagement.

We wish to add a further important caveat here. If someone has lived a life that has been particularly perverse by any reasonable standard, then this may constrain one’s capacity for even interpretive self-authorship yet alone agential self-authorship. We mentioned earlier that the events of one’s life do place certain constraints on the kind of meaning that one can construct from one’s life. Thus, if someone has lived a particularly evil or dissipated life, one’s capacities to draw positive meaning from one’s life would be partially constrained. The Roman emperor Nero is purported on his deathbed to have uttered the words, *“Qualis artifex pereo”* (“what an artist dies in me”) but it is hard to see Nero’s life as anything but that of a murderous dictator. Nevertheless, even in these circumstances, the end of life may still provide an opportunity for repentance, apology, and reform, and for making whatever repair or reconciliation is possible, and possibly for finding some meaning in these.

Perhaps the processes of narrative consolidation may go some way in explaining some of the desires that people have to reconcile with loved ones before they die. Sometimes this can bring great peace to patients at the end of life. That being said, it seems that close social relationships matter in and of themselves and one would want to be careful about reducing their value simply to a higher order narrative significance they may occupy in a person’s life. It is to this topic of relationships that we now turn.

### Close social relationships

An important part of finding meaning and flourishing at the end of life are the relationships that one has. Part of this has to do with the fact that relationships can be important sources of wisdom for how one ought to live one’s own life. As Nussbaum and Levmore write in *Ageing Thoughtfully*,

we can learn from each other's experiences…when an isolated individual observes and contemplates, it is hard to discern whether one has become more self-absorbed, more accepting of criticism, more frightening to others, or more unreasonable in making demands on family members. Self-knowledge might therefore require friendships and conversations [44, p. 2].

And, we might add, the flourishing of each individual might require close social relationships that allow one to better understand the extent to which one is in fact flourishing or languishing. Friendship is a means by which one can contemplate virtue and better understand it, according to Aristotle: “We can contemplate our neighbors better than ourselves and their actions better than our own” [13, IX.9].

But close social relationships are valuable in and of themselves and plausibly essential constituents of the good life. Brad Hooker defines deep personal relationships as those that involve “deep mutual understanding and strong mutual affection” [9, p. 1]. Aristotle famously argued true friendship is rare, in part because it takes time to become accustomed to each other and “appear loveable” to the other [13, p. 1156b25-35]. Certainly, one could only have a limited number of deep personal relationships in one’s life given the time and affective investment that these relationships require. But they are a vital part of the good life. Thus, Aristotle writes that it is better to spend one’s days “with friends and good men than with strangers or any chance persons” [13, IX.9]. More fundamentally, “no one would choose the whole world on condition of being alone, since man is a political creature and one whose nature is to live with others” [13, IX.9]. Cicero in *De Amicitia* goes as far to argue that a friend is a kind of alter-ego that gives one “a second life:”

In the face of a true friend a man sees a second self. So that where his friend is he is; if his friend be rich, he is not poor; though he be weak, his friend's strength is his; and in his friend’s life he enjoys a second life after his own is finished [45, no. 7].

This is quite a profound consideration in an end of life context given that patients often think about what shall remain of them and their legacy when they pass away.

How, then, might deep personal relationships be cultivated in a unique way at the end of life? Certainly, terminal illness typically generates sympathy from one’s loved ones and appreciation both on the part of the patient and their loved ones that the remaining moments spent together are particularly important and pregnant with meaning. People are willing to ‘drop everything’ to come see a loved one at the end of life. The time spent together may take the form of a review of the joys and woes of the dying person’s life and a celebration of the relationships that one has with their family and friends. Assurances may be made between patients and their families at the time of death of accompaniment and solidarity in the dying process, and the physical gestures that one typically witnesses in the context of dying (e.g., the holding of hands, the stroking of a loved one’s cheek, and so forth) denote loving accompaniment. Even if a patient is unable to speak, the fact that they are accompanied by their loved ones may mean just as much if not more than consoling conversations.

Granted, some may feel deeply alone at the end of life. Illness as such is isolating, and this is particularly true of terminal illness. But a deepening of intimate relationships is paradoxically facilitated in some cases by the isolation of illness. In other cases, illness might itself result in the offer of much greater social support. The end of life can also, for example, be a time for rapprochement between estranged friends and family.

In end of life contexts a unique bond can also be forged between caregivers and patients. Hospitality has been characterized as involving a communication of “something of oneself to the other” [46, p. 91]. Often it will be someone’s immediate family who is most directly accompanying a patient in illness. But sometimes clinicians can be part of this, too. This may present a difficulty for the profession of palliative care. Palliative care may require a deeper emotional engagement that other areas of medicine may not, though studies appear to suggest that palliative care physicians do not experience higher rates of burnout than other medical specialties [47].

Consider also that death is sometimes the horizon against which relationship commitments are made. Some marital vows, for example, may be predicated on a commitment to fidelity until ‘death do us part.’ One definitively fulfils these commitments at the moment of death and one’s relationship thus takes on new contours, at once mournful and beautiful, as one approaches death. But even less explicit commitments, such as loving someone *forever*, implies death as a horizon. As such, there is a sense in which relationships are brought to full completion at the end of life and the virtue of fidelity is fully realized.

Much more could be said about relationships and social connectedness in the context of terminal illness, but suffice it to say that there is a unique significance about the relationships that one maintains and cultivates in the context of terminal illness. Indeed, one can become more deeply connected with others than at any other time in one’s life. Relationship commitments are also often definitively fulfilled in the context of death in such a way that one’s relationships take on an additional depth at the time of death.

### Virtue and character

Some of the discussion so far has already highlighted how the end of life may afford people the opportunity to grow in character and virtue. Character is sometimes defined as “a set of personal traits or dispositions that produce specific moral emotions, inform motivation and guide conduct” [48, p. 2]. Bernard Williams has also offered a definition of character in terms of the life goals or ground projects to which an agent is committed [49]. Virtue has been defined in terms of stable dispositions of a person that orient them toward the pursuit of the good. Aristotle distinguished between intellectual and moral virtues, but it is important to note that virtues engage not just the mind but also the will and emotions of a human person. There has been a revival of interest in virtue ethics in recent decades, but perhaps less emphasis on the cultivation of virtue and character in end of life contexts. This may be related to cultural denial of death and dying in modern Western culture [11].

Death is a moral and existential challenge, and, according to at least some religious traditions, the ultimate challenge to one’s moral character. Several common prayers in the Catholic tradition, for example, focus on the theme of final perseverance at the time of death [50]. This preoccupation does not seem entirely misplaced. At the end of life, one’s body is literally ‘disintegrating.’ The intricately integrated biological systems of the body are ceasing to work together. There is also disunion between the body and one’s psychology. A non-ambulatory patient, for example, may want to get up and walk around in the garden, but their body will not allow them to do so. The intactness of the person is threatened in the most profound of ways when one is suffering from the effects of a terminal illness [51].

As such, the opportunities for despair, resentment, unhelpful cycles of self-pity, and a closing off of oneself from others are myriad at the end of life. Paradoxically, however, this challenge can facilitate a growth in character and virtue. Virtues, after all, are acquired through practice, particularly practice in challenging situations. If one did not have adequate occasion to exercise virtue, then one would not be able to acquire virtue.

There are many virtues that appear to be tested at the end of life. But drawing upon the inner logic of the *Ars Morendi* literature, we would give particular emphasis to the virtues of patience, hope, humility, and faith [11, 52, 53]. It would be beyond the purposes of this paper to highlight in detail how each of these virtues is put to the test at the end of life. But clearly when one is faced with the profound suffering, uncertainty, disempowerment, and isolation that are typically felt in the final chapter of life, one needs to exercise virtues that allow one to come to terms with the human condition (and, by doing so, that allow one to transcend the finitude of one’s human condition). If human fulfilment consists in rational activity in accord with virtue as Aristotle thought, then the end of life provides a difficult, but potentially enriching, context in which to realize that potential.

Are these virtues an unrealistic ideal for the end of life? Perhaps some might be tempted to think this. But anyone with experience caring for dying patients would know that people at the end of life can exhibit an amazing degree of selfless concern once they have come to terms with the fact that life is ending. We might also consider Tolstoy’s novella *The Death of Ivan Ilyich*—a story of a high-court judge in 19th-century Russia—Ivan Ilyich—and his suffering and death from a terminal illness [54]. Initially, Ivan’s psychological response to illness is, for the most part, one of despair and frustration at the interruption that it has caused to his otherwise simple, successful, and comfortable life. Ivan, however, reaches a turning point just hours from death at the novella’s very end. He cultivates a selfless concern for his wife and son and in doing so is able to overcome the existential pain of dying.

Some cancer patients, for their part, express a selfless concern for family in advanced care directives [55]. Often the biggest concern for a mother or father who is dying is not so much their own plight but rather the plight of their loved ones.

The very fact that many patients at the end of life are at peace with their situation is a remarkable expression of the virtue of contentment, which, while not part of the medieval *Ars Moriendi* tradition, does feature in the work of contemporary proponents of this tradition like Lydia Dugdale [53, 56]. We should not underestimate the loss of a sense of control and total dissolution of the ego that is involved in dying. To be able to face this experience with courage and serenity indicates strength of character and a deep appreciation of the fundamental goodness of the world despite the presence of profound suffering. Some philosophers argue that authentic human flourishing is in part dependent upon a recognition and virtuous engagement with the realities of our human condition [52, 53]. That is to say, for a human being to be flourishing, they must be living in a manner that is cognizant of and boldly accepting of the human condition. This includes accepting one’s mortality. Death can lead one to confront the fact of one’s mortality in the most real and virtuous way one can: by living in bold acceptance of it.

Building on this insight, part of the work of palliative care involves helping patients to live with the tension of trying to be engaged in life while accepting that they are approaching death. Palliative care specialists can guide patients such that they can be more mentally and emotionally flexible and predisposed to accept death as a normal part of life [57, 58].

## Summary

These considerations go at least some way in illustrating the different ways in which people can be said to flourish at the end of life in the context of rapidly declining health and other challenges that accompany the dying process. There may, in fact, be other goods that people realize at the end of life that we have not included in our discussion. But for the sake of brevity we have limited our discussion core aspects.

Notwithstanding the human drama of suffering, the possibility remains open that someone can flourish both despite the suffering accompanying terminal illness and even in some cases by adopting a transformative approach toward it. This may require that theorists of wellbeing take seriously the possibility that patients can adopt a transformative approach to suffering, whereby suffering is viewed as an occasion for growth of the person rather than just something that one seeks to remove or merely endure [52, 59]. By a transformative approach to suffering we have in mind a response to suffering that intentionally seeks personal growth and meaning by means of the suffering itself, or the approaching of the end of life. We have articulated an account of narrative consolidation, close social relationships, and the cultivation of virtue and character whereby terminal illness allows for and facilitates the unique realization of these goods. This is different from approaches that focus on tolerating or removing the suffering. We are not seeking to present an alternative coping strategy but rather a completely different, though possibly also complementary, response to the suffering brought by terminal illness and approaching the end of life [21]. Additionally, the work of Viktor Frankl as well as Bowker’s work on religious perspectives on suffering can be helpful resources for this task [60, 61].

We conclude from these considerations that there is a qualified sense in which one can be said to flourish at the end of life, notwithstanding the complexities of deploying this construct in end of life contexts. Particularly striking is the profound variability in the way people respond to their own impending death. This is one reason for focusing on meaning and purpose understood as narrative integration as well as close social relationships and virtue. These three aspects of a person’s life are particularly relevant to how one responds to the ‘existential slap’ of terminal illness [62]. If one has close friends and family and a capacity for virtue and narrative integration, one will be more likely to positively respond to various existential challenges that arise at the end of life. These existential challenges, in turn, will be more likely to facilitate a deepening in familial and fraternal relationships, virtue and character, and narrative integration.

## Current methods for assessing wellbeing at the end of life

There are several validated quality of life scales to measure wellbeing at the end of life. These measures focus on domains that are thought to be central to the preferences and life satisfaction of patients at the end of life. These include symptom control, physical function, independence, psychological wellbeing, and social relationships [63]. To be clear, we are focusing here on measures commonly employed in palliative care, and this is not intended as a review of wellbeing assessment tools in other domains such as gerontology. Considerations of gerontology and aging extend beyond end of life settings; additionally, end of life settings may sometimes be faced by those who are young.

Perhaps the most comprehensive survey tool for end of life care contexts is the McGill Quality of Life Questionnaire [64], which has been revised and expanded in recent years [65]. The McGill QOL questionnaire aims to assess multiple domains to identify end-of-life quality. These domains include physical symptoms, psychological and existential well-being, social environment, cognition, quality of healthcare, and one’s sense of being a burden on others. Respondents are asked to answer questions on a 0 to 10 scale. The MQOL and the MQOL-revised questionnaire allow respondents to list their most problematic physical symptoms rather than providing them with a list of symptoms that may not accurately represent their physical condition.

There are several other Quality of Life measures, such as the European Organization for Research and Treatment of Cancer QLQ-C15-PAL, which utilizes the presence of multiple symptoms to assess quality of life in palliative care contexts [66]. But the McGill Quality of Life questionnaire seeks to assess the subjective impact of symptoms on quality of life rather than taking the symptoms to be themselves solely constitutive of quality of life. The McGill questionnaire also includes existential and spiritual domains and, in the expanded questionnaire, several other psychosocial domains. A close cousin of the McGill Quality of Life survey is Chochinov’s patient dignity inventory, which seeks to “measure various sources of dignity-related distress among patients nearing the end of life” [67, p. 559]. Like the McGill questionnaire, Chochinov’s survey considers not just the immediate physical and psychological burden of illness but also the extent to which patients are able to live a meaningful and fulfilled life when sick.

In addition to quality-of-life questionnaires for patients in palliative care, researchers have also developed death acceptance and anxiety scales and good death inventories which can help assess the extent to which patients, family, and clinicians are comfortable as death approaches or once death occurs. A death acceptance scale seeks to assess the extent to which someone is comfortable and at peace with the reality of death in general and one’s own future death in particular. These surveys are not necessarily designed specifically for palliative patients but could be employed in this context [68]. A death anxiety scale is a psychological tool that is used to measure an individual's level of anxiety or fear related to death and mortality (see, for example [69]). It aims to assess the intensity and impact of death-related anxiety on an individual's psychological well-being. This type of scale helps researchers and clinicians understand how individuals experience and cope with anxiety about death. Finally, a good death inventory assesses the extent to which a patient’s family and friends feel that a patient has died a good death. The core domains of one such inventory include ‘‘environmental comfort,’’ ‘‘life completion,’’ ‘‘dying in a favorite place,’’ ‘‘maintaining hope and pleasure,’’ ‘‘independence,’’ ‘‘physical and psychological comfort,’’ ‘‘good relationship with medical staff,’’ ‘‘not being a burden to others,’’ ‘‘good relationship with family,’’ and ‘‘being respected as an individual” [70, p. 613].

One limitation we have identified with these survey tools is that their focus appears to be on coping with terminal illness rather than holistic wellbeing at the end of life. The Miyashita good death inventory affirms independence of others as a feature of a good death and this notion would need clarification to render it compatible with our account of flourishing (notwithstanding the fact that many end of life patients value independence in carrying out everyday activities). The McGill QoL questionnaire and Chochinov’s Patient Dignity Inventory are, perhaps, exceptions. These two survey tools adopt a more holistic approach to wellbeing and consider factors such as how one feels about one’s life or the extent to which one feels supported by a community of friends and family. Even still, their ultimate focus is different from flourishing insofar as they focus rather on quality of life or dignity at a particular moment in one’s life. On at least some accounts, quality of life is a subjective measure of wellbeing concerned with an individual’s perception of their position in life with respect to their goals, expectations, standards, and concerns [71]. Also there, dignity focuses on the sense of self-worth and respect that a person experiences [72]. These constructs are distinct from flourishing insofar as they focus more on time-slice, subjective satisfaction, or a sense of respect and self-worth. These attributes are not irrelevant to flourishing, but flourishing goes beyond subjective satisfaction at a particular point; it also transcends self-worth and a sense of respect and concern from others, vitally important though these things may be.

Related to this, these survey tools do not emphasize virtue and character. As such, these tools seem to omit key domains of wellbeing that feature and are central in classical and many contemporary accounts of human flourishing [52, 59] and in our discussion above.

There may, of course, be a reason why virtue and character are not assessed in existing survey tools. This could be the assumption that we identified at the start of this paper, namely, that flourishing is not possible at the end of life and that the use of this construct is inappropriate in the assessment of wellbeing at the end of life. However, as argued above, we believe this assumption is incorrect. We thus now turn to consideration of a possible measure of flourishing at the end of life.

## The assessment of flourishing at the end of life

If flourishing is indeed possible at the end of life, what implications does this have for the assessment of wellbeing? The indices of existing Quality of Life scales reflect the fact that various features of terminal illness—such as that the time horizon on one’s life is so limited—mean that there is a reorientation of one’s life goals. As such, one may ask the question whether modifying our current approach to conceptualizing and assessing wellbeing at the end-of-life is even necessary.

There is, however, a conceptual ceiling to existing measures of wellbeing at the end of life which seems to center on the notion of peace and comfort understood as the absence of psychological distress and physical pain. This limited conception of the horizons of wellbeing at the end of life does not give due recognition to the full potential for meaning, close relationships, and growth in virtue at the end of life. One can contrast this with the *Ars Moriendi* literature which seems to portray one’s dying days as the most existentially significant moment in one’s life. To reiterate, we are not trying to say that lifetime wellbeing is solely about how one spends one final days; but these final days are, nevertheless, highly significant from a moral, existential, and spiritual perspective.

Perhaps, then, one ought to use a flourishing assessment for palliative care and end-of-life contexts that considers both profound limits as well as vast possibilities for spiritual, moral, and social growth that at least some people experience at the end of life. We noted how existing quality of life scales for end of life settings are very sensitive to the unique preferences that come to the fore for patients at the end of life. For example, some surveys focus on a *capacity for independent living* or *vitality*—indices that are particularly relevant for people suffering from illnesses that often if not typically result in disability. The same context sensitivity could be reflected in surveys used to measure human flourishing. We contend that flourishing in this context ought not be assessed in terms of one’s possession of good health nor solely in terms of quality of life but also with a strong reference to human goods such as meaning and purpose and deep personal relationships. Certainly, one should not write off one’s capacity for flourishing at the end of life simply because one is in a state of poor health or has reduced quality of life.

In the “Appendix” we have put forward an adaptation of VanderWeele’s flourishing index [8], altered for end-of-life contexts. The original assessment was intended for general use in adult populations and assesses well-being with twelve questions across six domains: happiness, self-rated health, meaning, character, relationships, and financial stability. Unlike many other well-being assessments, it includes a domain for character and virtue, which, as argued above, is especially important in end-of-life contexts. The length of the original assessment is sufficiently short that it has been used in a wide variety of contexts [73, 74]. Further information on the measure itself and its psychometric properties across settings and cultures is available elsewhere [74, 75].

The adaptation employs the same six domains as the original flourishing assessment but alters some of the specific questions to take into account the end of life setting. The assessment’s physical health question is replaced by a question about freedom from physical pain. The question on understanding *purpose* in life is modified to constitute a question on understanding the *meaning* of one’s life, in light of the shift from ‘agentive’ to ‘interpretative’ self-authorship. The question on assessing whether one’s activities are worthwhile is modified to concern the extent to which one’s past activities in life as a whole are being understood as worthwhile. The question on delayed gratification is replaced by one on opportunities for personal growth. The question on satisfying relationships is modified to focus on the final stages of those relationships. The questions on happiness and financial stability are the same.

In prior use of the flourishing assessment, outside of end of life contexts, the domains have often been reported separately since dynamics can be quite different across the domains. We would recommend the same here in end of life contexts. We would also recommend placing special emphasis on the meaning, relationships, and character domains in end-of-life settings. We believe the other domains (happiness, health, financial security) are worth assessing as well, principally to try to understand whether there are practical needs (e.g. concerning pain, anxiety, despair, financial worries, etc.) that need to be addressed. However, since the domains of meaning, relationships, and character might be taken as primary at the end of life, perhaps these three domain scores could even be averaged as a crude assessment of flourishing at the end of life; that is to say, flourishing conditional on the end of life context, acknowledging that health is in decline and that life is reaching its end.

The modifications suggested here have been put forward so that end of life flourishing assessments domains correspond to the more generic flourishing assessment [8] but are more appropriate to the end of life stage. An analogous adaptation has likewise been made for adolescent flourishing acknowledging the adolescent life and developmental stage [40, 76]. Future work could evaluate psychometric properties of this end of life adaptation and consider its relations to the more general flourishing assessment and the differences and limitations concerning the use of the general flourishing assessment in end-of-life settings. It would likewise be desirable to supplement this basic end of life flourishing assessment with additional assessments related to (i) a patient’s specific condition, and/or (ii) care for the specific spiritual or religious concerns a patient may be encountering [77]. Disease-specific quality-of-life assessments—that are sometimes more focused on particular symptoms and challenges characteristic of a given condition or disease—could be viewed as complementary, rather than as an alternative, to these broader end of life flourishing assessments. Similarly, the use of these questions in the context of patient care could be viewed as complementary, rather than as an alternative, to more general open-ended questions. The asking of specific questions concerning flourishing may open the door for patients to raise issues that either do not immediately spring to mind, or that patients may be otherwise hesitant to address. Spiritual care at the end of life, which may depend on a particular religious tradition, may likewise also be viewed as complementary rather than as an alternative to these more universal flourishing assessments.

Some limitations of the proposed questionnaire ought to be noted. First, patients with dementia may be unable to complete the questionnaire depending on the severity of their condition, or alternatively, may find it quite taxing. This is a limitation that other end of life quality of life surveys, such as Chochinov’s Patient Dignity Inventory, also face [78]. Second, one would need to consider how fluctuations in mood and cognitive capacity during the day might affect the way in which respondents complete the survey. Factors such as transient pain can substantially alter responses and may lead to results that do not reflect the more stable, or at least average or typical, assessments a patient might give. It may be necessary to administer the survey multiple times over the course of several days or weeks to get an accurate representation of a patient’s flourishing. Third, some prospective respondents may not have come to terms with the fact that their life is ending, or they may have unrealistic hope of recovery from their terminal illness. Clinicians would need to be mindful of these factors when considering who to approach about completing such a flourishing survey.

## Conclusion

This paper has argued that human flourishing is possible in a qualified way at the end of life, and that one ought to adjust one’s understanding of flourishing in this context to be sensitive to goods that are uniquely realizable when one is dying. In light of our argument, we encourage further empirical study of the diverse ways in which people can realize the goods of meaning, close social bonds, and virtue and character at the end of life. With appropriate empirical evidence, clinicians would be well-placed to offer interventions that might help people nearing the end of life better realize these goods and attain higher levels of wellbeing in their final days and weeks of life. Such considerations may also come into play in discourse and discussion concerning euthanasia and physician-assisted suicide. Decision-making and evaluation may appear rather different when considerations of meaning, character, and relationships are taken into account, as compared to when there is a more exclusive focus on life satisfaction or financial stability, which are often dominant.

At the very least, we hope to have shown that terminal illness is indeed compatible with the possibility of flourishing in a qualified sense, and that there may be significant opportunities for existential and moral growth even when one is in one’s final days of life. It is our hope that this paper will contribute to a more nuanced understanding of the dynamic character of human flourishing. In addition to considering what it means to *live well*, the discipline of wellbeing studies ought also to turn its attention to what it means to *die well*.

Other future directions of research might include interventions that can help people to flourish at the end of life. Some examples that are already present are dignity therapy [79], legacy project interventions [80], spiritual care interventions [81], and forgiveness interventions and relationship counselling [82]. Rather than any one intervention being uniquely conducive to flourishing, a combination approach would take into account the specific circumstances of each patient. It may, nevertheless, be possible to greatly enhance the possibilities for flourishing at the end of life given appropriate interventions.

Donaldson has argued that clinicians ought to “mak[e] the promotion of human flourishing in the context of health the guiding goal for medicine”[12, p. 4]. Clinicians and patients in acute and end of life settings certainly also stand to benefit from adopting a broader lens on the goals of care—one that seeks to promote human flourishing even when life is threatened or ending [58].

## Appendix: End-of-life flourishing assessment

Please respond to the following questions on a scale from 0 to 10:

1. Overall, how satisfied are you with life as a whole these days?
  -         0 = Not Satisfied at All, 10 = Completely Satisfied.
2. In general, how happy or unhappy do you usually feel?
  -         0 = Extremely Unhappy, 10 = Extremely Happy.
3. To what extent have you been free of physical pain this past week?
  -         0 = Intense Pain, 10 = Completely Free of Pain.
4. How would you rate your overall mental health?
  -         0 = Poor, 10 = Excellent.
5. Overall, to what extent do you think the things you have done in your life were worthwhile?
  -         0 = Not at All Worthwhile, 10 = Completely Worthwhile.
6. I understand the meaning of my life.
  -         0 = Strongly Disagree, 10 = Strongly Agree.
7. I always act to promote good in all circumstances, even in difficult and challenging situations.
  -         0 = Not True of Me, 10 = Completely True of Me.
8. Even now, I am finding opportunities for personal growth.
  -         0 = Not True of Me, 10 = Completely True of Me.
9. I am content with my friendships and relationships.
  -         0 = Strongly Disagree, 10 = Strongly Agree.
10. The final stages of my relationships are as satisfying as I would want them to be.
  -         0 = Strongly Disagree, 10 = Strongly Agree.
11. How often do you worry about being able to meet normal monthly living expenses?
  -         0 = Worry All of the Time, 10 = Do Not Ever Worry.
12. How often do you worry about safety, food, or housing?
  -         0 = Worry All of the Time, 10 = Do Not Ever Worry.

Item 3 replaces the original “In general, how would you rate your physical health?” [8, p. 8154]. Item 5 is a modification of the original “Overall, to what extent do you feel the things you do in your life are worthwhile?” [8, p. 8154]. Item 6 is a modification of the original “I understand my purpose in life” [8, p. 8154]. Item 8 replaces “I am always able to give up some happiness now for greater happiness later” [8, p. 8154]. And item 10 is a modification of the original “My relationships are as satisfying as I would want them to be” [8, p. 8154].

## Footnotes

1 Notably, Harvey Chochinov's Dignity Therapy (therapy for the end of life stage) [28, 29] has as its primary goal the exploration of a narrative of the arc of a person's life, and has been tested in palliative care and hospice settings.

2 Another potential limitation may arise in the context of pediatric terminal illness where the limited maturity and life experience of a child may limit their capacity for narrative consolidation and the cultivation of virtues relevant to dying well. We simply note this limitation and will not here engage specifically with problems of applicability to pediatric populations.

3 See [39] for an account of the effect of narrative construction on wellbeing.
