# Correction: Flourishing at the end of life

**DOI:** 10.1007/s11017-024-09691-1

**Published:** 2024-10-19

**Authors:** Xavier Symons, John Rhee, Anthony Tanous, Tracy Balboni, Tyler J. VanderWeele

**Affiliations:** 1 Human Flourishing Program in the Institute for Quantitative Social Science, Harvard University , Cambridge, MA USA; 2 Plunkett Centre for Ethics, The Australian Catholic University, Sydney, NSW Australia; 3 Department of Epidemiology, TH Chan School of Public Health, Harvard University, Cambridge, MA USA; 4 Center for Neuro-oncology, Department of Medical Oncology, Dana Farber Cancer Institute, Boston, MA USA; 5 Division of Adult Palliative Care, Department of Psychosocial Oncology and Palliative Care, Dana Farber Cancer Institute, Boston, MA USA; 6 Department of Neurology, Harvard Medical School, Boston, MA USA; 7 School of Medicine, University of New South Wales, Sydney, NSW Australia; 8 Dana Faber Cancer Institute, Boston, MA USA

**Correction to: Theoretical Medicine and Bioethics (2024) 45:401–425** **https://doi.org/10.1007/s11017-024-09679-x**

In this article the affiliation details for Author John Rhee were incorrectly given as 'Department of Neurology, Dana Faber Cancer Institute, Boston, MA, USA' but should have been 1. Center for Neuro-oncology, Department of Medical Oncology, Dana Farber Cancer Institute, Boston, MA. 2. Division of Adult Palliative Care, Department of Psychosocial Oncology & Palliative Care, Dana Farber Cancer Institute, Boston, MA. 3. Department of Neurology, Harvard Medical School, Boston, MA.

The original article has been corrected.

